# Case of Areolar Cancer of the Peritoneum

**Published:** 1861-01

**Authors:** J. Forsyth Meigs

**Affiliations:** Physician to the Pennsylvania Hospital


ART. III.—*Case of Areolar Cancer of the Peritoneum, with Hæmatocele of the right and Encephaloid Disease of the left Ovary.* By J. FORSYTH MEIGS, M.D., Physician to the Pennsylvania Hospital.

MISS B., aged twenty-seven, was one of a large family, the members of which are generally healthy, with the exception of one sister, who has had several attacks of slight hæmoptysis, but who is still living in good health. Her mother died, when Miss B. was still young, of phthisis. Her own health had been good, with the exception of an attack of typhoid fever and some suffering from dysmenorrhœa, up to the spring of 1860, when she began to lose color, and complained occasionally of not feeling quite so strong as usual, but was not thought by her family to be seriously ailing, and consulted no physician, as she would certainly have done had either she or her family supposed that she was seriously out of health.

About the middle of June, 1860, toward the menstrual epoch, she helped to carry a moderately large trunk down the staircase. She had taken the lower end of the trunk in order to save a favorite elderly servant, and bore the principal part of the weight. While doing this she was seized suddenly with a pain, accompanied by a sensation of a snap, as of something giving way, in the region of the left groin. This pain was quite severe at the moment, but soon passed away, and was entirely forgotten until recalled by careful questioning as to the cause of her illness six weeks afterward. The night of the day on which this occurred she had some nausea, and was not well from that time.

About the seventh of July I was sent for to see her. She was at a friend's house, going about as usual, not thinking herself seriously ill. I was told that she had pain in the lower part of the back, in the groins, and hypogastric region, not severe, but annoying. She had frequent micturition, severe pain in voiding the bladder, decided constipation, and considerable nausea. Her appearance was that of one suffering from moderate anæmia. I supposed that she had some displacement of the uterus, and ordered a mild cathartic composed of senna electuary, cream of tartar, sulphur, and carbonate iron, (Graves's paste,) an elixir of cinchona, and a great deal of rest on the back.

I saw her again on the ninth of July, at her window, dressed, in excellent spirits, merely looking rather pale, and with the same symptoms as before, except that the pain had increased.

I left town on the following day, and did not return until the twenty-fourth of July.

A few days after this her symptoms became much more severe, and Dr. F. G. Smith was sent for. She now had severe pain in the pelvis, especially about the left groin, and pain in passing water, without, however, very frequent desire. The most marked symptom was constipation, and inflation, with hardness, of the abdomen. About the sixteenth of July, Dr. Smith discovered a hard tumor, pyriform in shape, about the size of a small pear, jutting up apparently from the pelvis, somewhat to the left of the symphysis pubis. Castor-oil and some other cathartics were given. These operated moderately. She suffered severely, especially in paroxysms, which were attended with nausea, and sometimes with vomiting. The abdomen was markedly distended all over, and sonorous; very little flatus escaped per anum, and she never felt thoroughly relieved by the stools. Had opiate suppositories every night, without which she could not sleep for pain.

On July twenty-fourth I took charge of the patient again. She looked badly, pale and thin, with a face expressive of suffering. Abdomen full, distended, and sonorous on percussion, all over; below, tender to the touch, particularly toward the left groin. Low down, between the symphysis pubis and left Poupart's ligament, could be felt, rather indistinctly, a somewhat pyriform tumor, which was hard and tender to touch. In this region, also, could be detected an obscure fluctuation.

Finding that the principal symptoms were those of obstructed bowel, depending apparently on the pressure of some growth or deposit in the left pelvic region, cathartics were administered. A pill of blue mass, colocynth, and aloes was tried, but could not be retained. Small doses of the species St. Germain were then given every four hours, and were borne very well. Enemata of flaxseed tea was also given, and at first these operated somewhat, bringing away dark-brown, healthy feculent matter in small quantities, and of the consistence of mush. Only once was there any solid matter observed, and this was composed of a few small, feculent lumps. At this time no flatus was passed, though she felt as though this might be all that was necessary for her relief.

She had now occasional severe pains throughout the abdomen, of a griping character, with much nausea, and with vomiting from time to time. Much soreness in left iliac fossa and in the hypogastric region.

On the 28th, a per vaginam examination was made by Dr. C. D. Meigs.

The uterus was ascertained to be situated low down in the pelvis, and to be immovable, but there was no retroversion, no tumor, nor any fluctuation in the vaginal walls. At this time the whole abdomen was greatly distended; it was tight, tense, loudly resonant above the umbilicus and in the right iliac fossa, while just above the symphysis pubis, and in the left iliac fossa, it was dull, and with a marked sense of fluctuation.

She now grew rapidly worse, and died, after great suffering, on the morning of August 4th.

During the last five days of her life she passed nothing from her bowels except a very little flatus now and then. From the 26th to the 28th of July, the pulse stood at about 140, small and weak; no heat of skin except a very little toward night; no chills nor creepings at any time. On the 28th and 29th July, the pulse fell to 128 and 120, after some good nights, procured by large opiate enemata, and the abdomen was rather less tense in the mornings.

On the 2d August, after a night of great suffering and prostration, Professor Gross saw her with me. At this time the general abdominal distention was very great. From the mesial line outward to the left, and as high upward as the left anterior superior spinous process, percussion was entirely dull, and here, also, there was perfectly distinct fluctuation. Elsewhere the abdomen was sonorous on percussion, and without any sense of fluctuation. Supposing that there might be pus, or an ovarian cyst in this region, we determined to make a puncture. Accordingly, at 5 P.M., Dr. Gross introduced a small trocar to a depth of about two inches, at a point two inches to the right of the anterior superior spinous process of the ilium, and a little below a line drawn horizontally from that process. From the canula there escaped about four quarts of a reddish, citrine-colored, serous liquid. This, on standing, deposited a layer, several lines in thickness, of blood, with a few very small coagula on the bottom and centre of the basin in which the fluid was placed. This fluid had a specific gravity of 1020, coagulated densely with heat and nitric acid, and exhibited under the microscope a considerable number of blood-globules, some of which had ragged, serrated margins; there were also a good many irregularly shaped hæmatoïdin crystals.

The operation gave very little pain. The abdomen fell at once to its natural size, without any escape of gas per anum, and now could be perceived in the left groin a hardened mass of irregular outline about as large as a small pear. At the time of the operation the pulse was 140. During the following night the pulse continued at about the same. The patient was very much exhausted, with collapsed features. There was no stool, but the urine was discharged without difficulty.

The next day there was no improvement. In after part of the day, severe pain set in again, which, in the course of the night, became violent in paroxysms. No stool. The night was passed in great misery, with frequent vomiting of a blackish fluid. Death occurred on the morning of the 4th, without any stool, and without any increase of size of the abdomen.

It should be stated that, during the last ten days, a very marked enlargement of the superficial veins of the left side of the abdomen had taken place. When this was first observed, there was no enlargement whatever of the veins of the right side, nor was there any swelling of the left leg. Five days before death there was decided enlargement of the veins of the left leg, with some swelling of the calf, and moderate œdema of the tissues over the left tibia.

As the fluid escaped from the canula, the net-work of enlarged veins on the left side of the abdomen disappeared.

The appetite was dull during the last three weeks, the tongue only moderately furred, and not dry. She took milk-punch, with lime-water and beef-tea. Various kinds of food and drinks were tried, but she liked nothing but mineral water, and for a short time, iced champagne.

The following account of the post-mortem examination was drawn up by Dr. S. W. Gross:—

The post-mortem examination was held on Sunday, August 5th, at 12 o'clock, and was confined to the abdominal and pelvic cavities.

The abdominal walls contained a slight amount of fat, and the body was not emaciated. On opening the cavity of the abdomen, the first fact that struck our attention was the presence of a large, blackish tumor, filling almost completely the pelvic cavity. It required some little force to lift it up, and it was found to be attached to the right broad ligament, its long diameter being placed in the axis of the pelvis, flattening the rectum posteriorly and tilting the uterus forward. It had the shape of the ovary, was five and three-fourths inches long, three and a half wide, and twelve inches in circumference. It was composed of organized clots of blood, which were evidently inclosed in the peritoneal covering of the ovary, the membrane being much thickened at the base of the tumor. The ovary lay imbedded in the mass, was about an inch and a half in length, and one in width, and was very vascular, bleeding freely when cut. There was no distinct line of demarkation between it and the clots. The pedicle was formed by the broad ligament, and was about three inches long. The os uteri was engorged, and the vesical and rectal reflexions of the peritoneum were greatly thickened. The left ovary had undergone cystic degeneration, forming a tumor four inches long, three wide, and nine in circumference. Its walls were greatly thickened and lobulated, and had undergone encephaloid degeneration.

The parietal portion of the peritoneum was almost universally the seat of cancerous tubercles, varying in size from that of a millet-seed to that of a small pea. The visceral portion of the membrane was not so much affected. The greatest seat of these masses was the great omentum, which was loaded with them, and was so much contracted as to be drawn up into the space between the transverse colon and the liver. The suspensory ligament of the liver, the gastro-hepatic omentum, and the mesentery were also greatly involved, and the pancreas was much indurated from the same deposit. The other abdominal organs were healthy.

The peritoneal cavity was circumscribed by adhesions which attached the cæcum, ascending colon, and small intestines to the posterior wall of the abdomen, and to so great an extent did these adhesions exist that the small bowel did not fall into the cavity of the pelvis. The abdominal and pelvic cavities contained about two quarts and a half of bloody serum.

Under the microscope, the tumors taken from the omentum and peritoneum exhibited fibrous tissue and cysts, inclosing simple and compound cancer cells and granules. The cancer juice, which was very abundant, also showed granules and compound granular cells, as well as well-marked and large caudate cells.

*Remarks.*—The case just described was evidently one of areolar cancer of the peritoneum, in which the peritoneal deposit assumed the appearance of "a hard, crystalline, transparent, and discrete cancerous follicle, resembling tubercle, and of the size of a hemp or millet seed," as it is described by Rokitansky.

The right ovary was greatly increased in size, as mentioned above, by a copious effusion of blood which had become coagulated between the outside of the stroma of the organ and the peritoneal investment. Was this an example of hæmatocele, occurring at the moment of the strain experienced by the patient? It is likely, also, that at the time the strain occurred, the ovary became dislocated into Douglas's cul-de-sac, and this gave rise, in part at least, to the obstruction of the intestine which was the immediate cause of death.

The left ovary exhibited an instance of the second variety of medullary carcinoma of the ovary described by Rokitansky, and which he says "is often, though not invariably, coexistent with areolar cancer of the ovary or of other organs."

An accurate diagnosis was not made during life. The opinion first entertained was that the case was one of pelvic cellulitis in the neighborhood of the left ovary, causing a tumor, with pressure on the rectum, and thus explaining the pain in the pelvis and the symptoms of ileus. Afterward it was thought that there might be a cyst of the left ovary, of very rapid formation, and to test this opinion, and also in view of the possibility that pus might be present, the puncture with the trocar was made.

It is easy to see now that the presence of the small hard tumor of the left ovary, and the subsequent rapid effusion into the left side of the peritoneal cavity, ought, perhaps, to have aroused suspicion of the existence of malignant disease of the peritoneum. But the strict limitation of this effusion in the region of the left iliac fossa seemed so adverse to the theory of peritoneal effusion, as to have prevented such an opinion from being formed. Moreover, the youth of the patient, and her previous very good health, both militated against any such opinion.

